# Indocyanine green excitation-emission matrix characterization: excitation-dependent emission shifts and application-specific spectra

**DOI:** 10.1117/1.JBO.31.7.077003

**Published:** 2026-07-29

**Authors:** Alberto J. Ruiz, Sophie A. Lyon, Ethan P. M. LaRochelle, Kimberley S. Samkoe

**Affiliations:** a QUEL Imaging, White River Junction, Vermont, United States; b Dartmouth College, Thayer School of Engineering, Hanover, New Hampshire, United States; c Dartmouth College, Geisel School of Medicine, Hanover, New Hampshire, United States

**Keywords:** indocyanine green, excitation-emission matrix, excitation-dependent fluorescence, red-edge excitation shift, anti-Kasha fluorescence, fluorescence-guided surgery

## Abstract

**Significance:**

Indocyanine green (ICG) is the most widely used fluorophore in fluorescence-guided surgery (FGS), and its microenvironment-dependent spectral response is relevant for system design, intersystem comparisons, and phantom development.

**Aim:**

To characterize ICG with excitation–emission matrices (EEMs) in the microenvironments of dimethyl sulfoxide (DMSO), bovine serum albumin (BSA) solutions, and 3D-printed (3DP) resin and assess excitation-dependent emission, including red-edge excitation shifts (REES) and departures from Kasha’s rule of excitation-independent emission.

**Approach:**

EEMs and absorbance spectra were acquired with extracted excitation spectra, emission spectra, emission peaks, centroids, and integrated emission areas under the curves (AUCs). Concentration-dependent behavior was examined in DMSO, and albumin concentration dependence was assessed from 5 to 100  mg/mL. Data processing employed robust local regression to mitigate excitation scattering artifacts.

**Results:**

ICG in DMSO exhibited excitation-independent emission consistent with Kasha–Vavilov behavior. By contrast, ICG in BSA solution and 3DP resin displayed excitation-dependent emission with pronounced REES and additional nonlinear departures from Kasha’s rule. To our knowledge, this represents the first documentation of REES and broader anti-Kasha effects for ICG or any FGS fluorophore. Within the excitation range most relevant to ICG-FGS (∼760 to 805 nm), emission spectra of the BSA solution and 3DP resin overlapped closely, with similar AUC-based comparisons, suggesting that ICG in 3DP resin can serve as a suitable surrogate reference for albumin-bound ICG.

**Conclusions:**

The EEM characterization shows that excitation-dependent behavior is a defining feature of ICG in biologically relevant environments, demonstrating that emission cannot be assumed to follow classical Kasha–Vavilov behavior. Reliable comparisons and imaging system design therefore require spectra acquired at defined excitation wavelengths with AUC integration within the emission detection band. Excitation-specific spectra from EEMs establish a consistent framework for intersystem comparisons and phantom standards, whereas the resulting datasets provide a practical spectroscopic reference for addressing excitation-dependent behavior in ICG sensing applications.

## 1 Introduction

Understanding the spectral characteristics of a fluorophore is crucial for fluorescence sensing,^1^ including fluorescence-guided surgery (FGS).^2^ The microenvironment of a fluorophore can significantly influence its physical and chemical behaviors, with factors such as local viscosity, polarity, temperature, redox conditions, and acidic-base status playing important roles.^3^ In fluorescence imaging, the most relevant changes associated with the fluorophore’s microenvironment are the excitation-emission spectral shifts and variations in the quantum yield,^4^ because they affect the sensitivity and specificity of fluorescence sensing devices.^5^^–^^8^ Among the microenvironment-sensitive spectral behaviors, solvatochromism reflects solvent-dependent differential stabilization of the ground and excited states and is observed as steady-state shifts in absorption and emission maxima.^9^^,^^10^ A complementary dynamic effect is the red-edge excitation shift (REES), in which the emission spectrum becomes excitation-wavelength dependent when solvent or protein relaxation occurs on timescales comparable to or slower than the fluorescence lifetime.^11^^–^^13^ This excitation dependence represents a departure from Kasha’s (Kasha-Vavilov) rule,^14^^,^^15^ which assumes excitation-independent emission for a given excited electronic state.^16^^–^^20^ In addition, excitation-dependent shift may be especially relevant in the complex environment of biological systems, where proteins such as serum albumin are present.^21^ As FGS continues to develop, comprehensive characterization of how fluorophore spectral properties respond to physiologically relevant microenvironments can help optimize optical system design, support inter-system comparisons, and guide the development of imaging phantoms and targets that support clinical translation.^7^^,^^22^^,^^23^

Indocyanine green (ICG) is the most widely used fluorophore in FGS, utilized for tissue perfusion, cardiac flow indication, and lymph node mapping.^24^^–^^26^ Its prominence in fluorescence guidance stems from its absorption and emission in the near-infrared (NIR) range, low toxicity, and extensive medical use for more than half a century.^24^^,^^25^^,^^27^^,^^28^ Understanding the variability of ICG’s spectral characteristics within *in vivo* environments can help optimize imaging system design and cross-system comparisons. This includes standard solvent effects as well as nonlinear shifts, such as REES. Solvatochromism of ICG has been widely studied and reported. ICG shows pronounced solvatochromism: solvent polarity and binding partners modulate its absorption/emission maxima and quantum yield; albumin binding typically red-shifts and stabilizes the spectra, whereas aqueous self-aggregation blue-shift absorption and quenches fluorescence.^29^^–^^34^ These environment-dependent shifts have been leveraged in various applications, including human serum albumin-ICG complexes to boost tumor-to-background contrast, conjugates for targeted imaging, liposomal and J-aggregate strategies to enhance stability/brightness, and solvatochromic readouts to map solvent composition and microenvironments *in situ*.^35^^–^^44^ Despite this extensive work on ICG solvatochromism, aggregation, and protein binding, we found no peer-reviewed reports documenting REES or other anti-Kasha excitation-dependent effects in ICG; in addition, comprehensive spectroscopy papers and clinical imaging reviews do not describe excitation-dependent emission consistent with REES or with departures from Kasha’s rule.^26^^,^^29^^–^^31^^,^^37^^,^^41^^,^^45^^–^^48^ By contrast, REES is well established in the literature for proteins, membranes, and protein–ligand complexes, where slow environmental relaxation relative to the fluorescence lifetime produces excitation-dependent red-shifts that can resolve microstate heterogeneity.^49^^–^^55^ Differences in excitation–emission behavior under varying environments can be captured by acquiring excitation–emission matrices (EEMs),^56^ which provide extended spectral information that support the assessment of solvatochromism, REES, and other microenvironmental effects.^12^

**Fig. 1:**
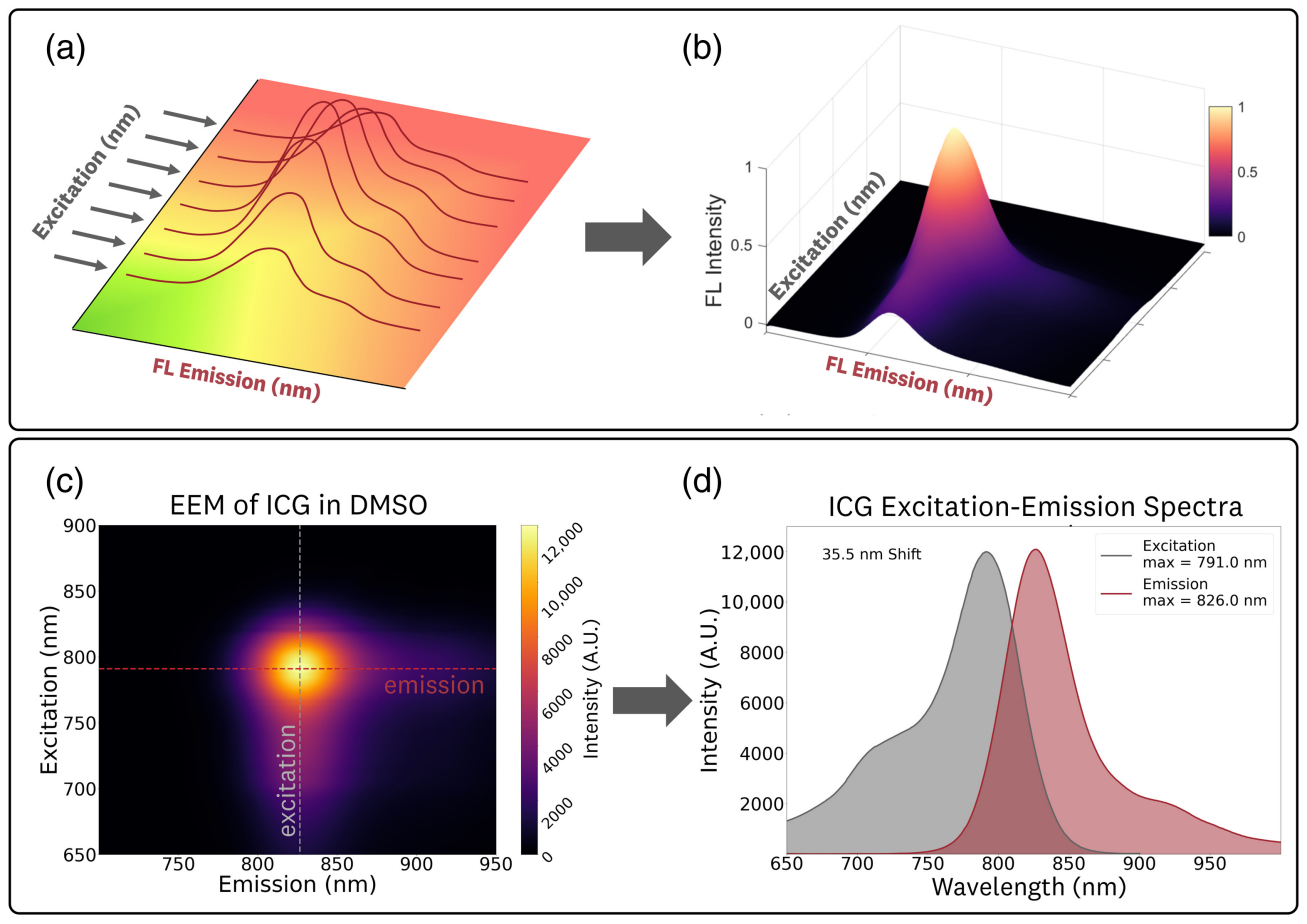
Excitation-emission matrix (EEM) of a fluorophore is generated by (a) acquisition of fluorescence emission spectra at various excitation wavelengths to produce the (b) three-dimensional EEM data set. The (c) EEM can be used to obtain conventional (d) excitation and emission spectra by isolating data along a single wavelength on the excitation or emission axis, respectively.

Excitation–emission matrices (EEMs) are three-dimensional datasets that map fluorescence intensity as a function of excitation and emission wavelength. They are typically acquired by recording emission spectra over a series of discrete excitation wavelengths [Figs. 1(a) and 1(b)]. Conceptually, an EEM can be viewed either as a stack of emission spectra at different excitation wavelengths or, equivalently, as a set of excitation spectra at fixed emission wavelengths [Figs. 1(c) and 1(d)]. This broad mapping of the excitation-emission properties provides more comprehensive information than conventional excitation-emission graphs collected at a single probing wavelength, enabling assessment of excitation-dependent phenomena such as REES. For fluorophores exhibiting excitation-dependent spectral shifts, EEMs allow derivation of emission spectra specific to the excitation characteristics of a given sensing system. A significant limitation of EEMs is the long acquisition times associated with obtaining fluorescence spectra across various excitation wavelengths. However, recent advances in CCD-based fluorescence spectrometers have enabled the rapid acquisition of these data sets. These developments provide a promising pathway for utilizing EEMs as a preferred alternative to individual spectra acquisitions, given their ability to characterize broader aspects of fluorophore behavior.

Furthermore, because FGS system performance can affect clinical decision-making,^5^^,^^6^ there is a compelling need for imaging standards that can help provide “ground truth” for system characterization.^7^^,^^57^ In this context, understanding how the excitation and emission of fluorescence imaging targets can mimic *in vivo* fluorescence is essential to provide relevant one-to-one equivalence to clinical fluorescence imaging applications.

Here, we report excitation–emission matrices of ICG in three microenvironments: dimethyl sulfoxide (DMSO), bovine serum albumin (BSA) solutions, and 3D-printed resin. These measurements establish comprehensive EEM characterization of ICG that reveal red-edge excitation shifts (REES) and departures from Kasha’s rule for ICG in serum albumin and 3D-printed resin, representing, to our knowledge, the first report of this phenomenon for ICG and for any FGS fluorophore. The EEM datasets are provided as supplementary files to support reproducibility, facilitate cross-system benchmarking, and serve as a reference for optical system development. Collectively, the results highlight new considerations for generating excitation-specific spectra, guiding fluorescence sensing system design and comparison, and informing phantom development for clinically relevant fluorescence imaging.

## 2 Methods

Preparation of the DMSO, BSA, and 3D-printed samples are described in Secs. 2.1–2.3. Procedures for the fluorescence EEM acquisition, absorbance measurements, and data processing are provided in Secs. 2.4–2.6. A complete list of the tested samples is provided in Table S1 of the Supplementary Material. The fluorescent samples were prepared using IR-125 laser dye (Exciton Inc., 09030), which is the laser dye marketing name of ICG (sharing the same Chemical Abstracts Service number, 3599-32-4), with identical chemical, quantum yield, and fluorescence spectral characteristics.^58^ IR-125 dye was utilized for this study, rather than clinical ICG dye, due to the availability of purity specifications from the respective manufacturers. Throughout the remainder of this study, the terms IR-125 and ICG are used synonymously.

### 2.1 Preparation of DMSO Solution Samples

EEM and absorbance measurements for the DMSO solutions were performed on standard 4.5 mL cuvettes (12×12×45  mm, 10 mm pathlength). A 1000  μM ICG stock solution was prepared in DMSO (Sigma-Aldrich, 472,301) to presuspend the fluorophore and minimize error during spectral comparisons.

For sample preparation, the 1000  μM stock was diluted with DMSO to yield 100 mL of a 1  μM working solution. A total of 3.5 mL of this 1  μM solution was transferred into a cuvette for data collection, with a matched control cuvette containing 3.5 mL of DMSO (0  μM ICG).

To study concentration-dependent EEM effects, an additional dilution of 100  μM was prepared from the 1000  μM DMSO stock. This 100  μM ICG stock was used to create, through serial dilution, 10 mL samples at concentrations of 10, 3, 1, 0.3, 0.1, and 0.03  μM of ICG in DMSO. Volumes of 3.5 mL of each of these solutions were deposited into cuvettes for data collection with an additional control cuvette of 3.5 mL of DMSO (0  μM ICG).

### 2.2 Preparation of BSA Solution Samples

EEM and absorbance measurements for the BSA solutions were performed on standard 4.5 mL cuvettes (12×12×45  mm, 10 mm pathlength). The 1  μM ICG in BSA solution (44  mg/mL) cuvette sample was prepared using the 1000  μM ICG in DMSO stock (Sec. 2.1), 150 mg/mL BSA stock, and phosphate-buffered saline (PBS). 10× concentrated PBS (Sigma-Aldrich, P7059) was diluted tenfold with distilled water to prepare 1× PBS. Lyophilized BSA powder (Sigma-Aldrich, A2153) was dissolved in the 1× PBS to the 150  mg/mL BSA stock. From these stocks, 100 mL of 1  μM ICG in BSA solution (44  mg/mL BSA) was prepared; 3.5 mL was transferred to a cuvette for data collection, with a matched control cuvette containing 3.5 mL of BSA solution (44  mg/mL, 0  μM ICG). The ICG in BSA solution was incubated at room temperature and protected from light for an hour prior to measurement. It should be noted that the 1000  μM ICG in DMSO stock solution (Sec. 2.1) was used in this preparation to mitigate the aggregation of dye in PBS solution, which was experimentally observed at these high concentrations. The 44  mg/mL BSA concentration was used as a representative concentration of albumin in human plasma.^59^^,^^60^

To study the effect of serum albumin concentration on spectral properties, 1  μM ICG samples were prepared at BSA concentrations of 5, 10, 25, 50, and 100  mg/mL. First, the 1000  μM ICG in DMSO stock (Sec. 2.1) was diluted with distilled water to prepare a 9  μM ICG aqueous stock. Separately, BSA was diluted with distilled water to generate two sets of 3.5 mL solutions at each target concentration (5 to 100 mg/mL): one set served as controls (0  μM ICG), and the other set received 389  μL of the 9  μM ICG stock to yield 3.5 mL cuvette samples at 1  μM ICG. Samples containing ICG were incubated at room temperature, protected from light, for 1 h prior to measurement.

### 2.3 Preparation of 3D-Printed Samples

The 1  μM ICG in 3D-printed (3DP) resin and the control (0  μM) samples were prepared using a proprietary clear photocurable resin (MML-REPC-001, QUEL Imaging) and the 1000  μM ICG in DMSO stock solution (Sec. 2.1). It is worth noting that, over the relevant 600 to 950 nm range, the clear resin has absorption and reduced scattering coefficient values of $\mu_{a}<0.003$ and $\mu_{s}^{\prime }<0.01\;{\mathrm{mm}}^{-1}$, respectively, as determined from integrating-sphere measurements using the inverse-adding doubling algorithm.^61^ The 3DP samples were prepared following a previously published method.^22^ In brief, 100  μL of the ICG stock was mixed into 100 mL of the clear resin and printed using layer-by-layer stereolithography printing (405 nm curing) to produce a fluorescent 3DP cuvette ($12\times 12\times 40\;{\mathrm{mm}}^{3}$) to match the dimensions of the standard cuvette samples; a control cuvette (0  μM ICG) was also printed using the same method. After printing, the cuvettes were cleaned with isopropyl alcohol and postcured utilizing a high-radiance 385 nm light (Solarez, 88,903). To minimize scattering of light, the 3DP cuvettes were progressively wet-sanded with distilled water and 1500, 2000, and 3000 grit silicon carbide sandpaper, followed by a two-step liquid polisher (Novus 7100). Sanding and polishing resulted in an optically clear surface, enabling data collection comparable to liquid samples prepared in a standard cuvette.

### 2.4 Fluorescence Excitation-Emission Matrix Acquisition

Fluorescence EEMs and absorbance spectra were collected using a dual fluorescence and absorbance spectrometer (Duetta, HORIBA Scientific). This spectrometer uses a xenon arc lamp with a monochromator, enabling excitation and absorbance sweeps over the 250 to 1000 nm range at 1 nm steps. It is equipped with a linear CCD sensor for fluorescence emission capture (250 to 1050 nm) and a silicon photodiode for absorbance measurements (250 to 1000 nm), utilizing right-angle (90 deg) excitation-emission geometry. For EEM acquisition, samples were excited from 650 to 900 nm in 1 nm increments, and the resulting fluorescence emission was collected over 600 to 1000 nm with a spectral resolution of ∼0.5  nm (1 pixel). All spectra were collected using a 3 nm excitation and emission bandpass slit size and three measurement accumulations. The reported maximum fluorescence emission values were assumed to have an uncertainty of ±1  nm, consistent with the manufacturer’s specified wavelength accuracy. Although the CCD provided a sampling resolution of ∼0.5  nm per pixel, the absolute calibration accuracy was ±1  nm and therefore taken as the limiting factor. Excitation values were likewise assumed to have ±1  nm uncertainty, based on acquisition step size and manufacturer specifications. The uncertainty of the reported Stokes shifts was assumed to be ±1.4  nm, obtained by adding the excitation and emission uncertainties in quadrature. Integration times of 1.0 to 2.0 s were used, with identical integration times maintained for comparative measurements to ensure consistent cross-sample fluorescence emission comparisons. For the varying concentration measurements of DMSO solutions (Sec. 2.1), the 3 and 10  μM samples were recorded with half the integration time to avoid detector saturation; their measured intensity counts were subsequently doubled during data processing to account for integration time differences.

The reported EEMs are calculated by subtracting the acquired data of the corresponding control (0  μM ICG) sample from the fluorescent sample. The emission spectra at a given excitation were obtained from plotting the intensity values along the emission axis of the EEM [Figs. 1(c) and 1(d)]. The excitation spectra at a “monitored” fluorescence wavelength were derived from plotting the intensity values along the excitation axis of the EEM [Figs. 1(c) and 1(d)]. The integrated fluorescence emission intensity at a given excitation wavelength, referred to as areas under the curves (AUCs) for the remainder of the paper, were calculated by trapezoidal numerical integration (implemented using the *trapz* function from the NumPy library) of extracted emission spectra (650 to 1000 nm), with emission centroids defined as the equal-area wavelength corresponding to the integrated distribution. Emission peak maxima were determined as the highest intensity values of the processed spectra (see Sec. 2.6 for details on data processing).

### 2.5 Absorbance Spectra Acquisition

Absorbance spectra were collected over the 600 to 1000 nm range at 1 nm step increments utilizing the spectrometer silicon photodiode detector (see Sec. 2.4 for instrument details). For all samples, an integration time of 0.1 s and a band pass slit size of 3 nm were used with corresponding blanks of DMSO, BSA, and 3DP resin. The reported max absorbance values are assumed to have a ±1  nm uncertainty, given the corresponding acquisition step size parameters and manufacturers’ wavelength accuracy specification. Absorbance data are reported with units of optical density (OD), where the measured $\text{transmission}=10^{\text{-}\mathrm{OD}}$. The manufacturer provides an absorbance accuracy specification of ±0.02  OD.

### 2.6 Data Processing

**Fig. 2:**
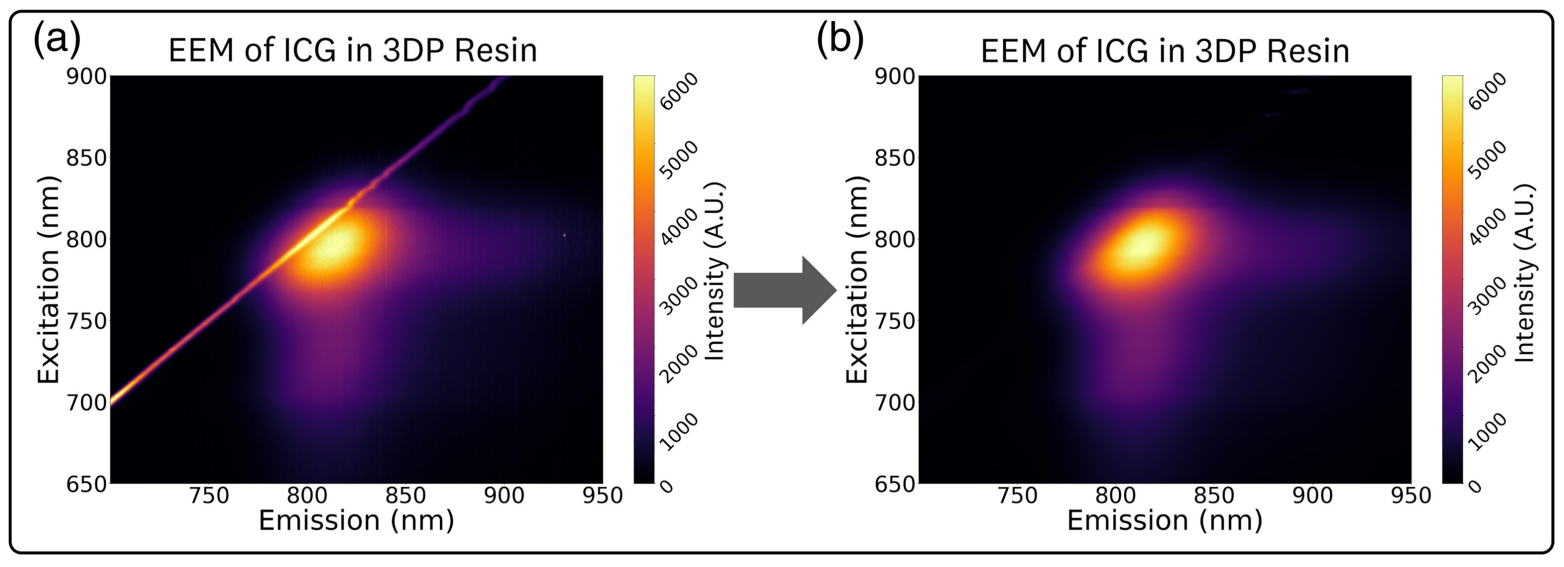
EEMs for samples with inherent scattering (3DP resin and albumin solutions) exhibit a scattering peak along the excitation-emission line (a), which can be corrected through the use of the RLOESS smoothing algorithm to result in the post-processed EEMs (b).

The EEM and absorbance spectra were processed using robust local regression smoothing with a span of 40 nm. The particular method utilized, robust locally estimated scatterplot smoothing (RLOESS), uses robust locally weighted linear least squares regression with a second degree polynomial model.^62^^,^^63^ This robust smoothing allows for the elimination of outlier measurements and generally provided better peak estimations than traditional smoothing techniques (i.e., Savitzky-Golay filtering) when tested against Gaussian and skewed Gaussian fits to the spectral data. Furthermore, this robust smoothing provided the ability to eliminate scattering peaks introduced by the BSA and 3D printed resin. An example of this scattering artifact elimination by the robust smoothing is shown in Fig. 2. RLOESS can be implemented through the use the *smooth* function in MATLAB or, equivalently, in Python with a modified implementation of the ^64^ *loess* project,^65^^,^^66^ which provides equivalent robust weights to the native MATLAB implementation.

## 3 Results

### 3.1 ICG in DMSO

**Fig. 3:**
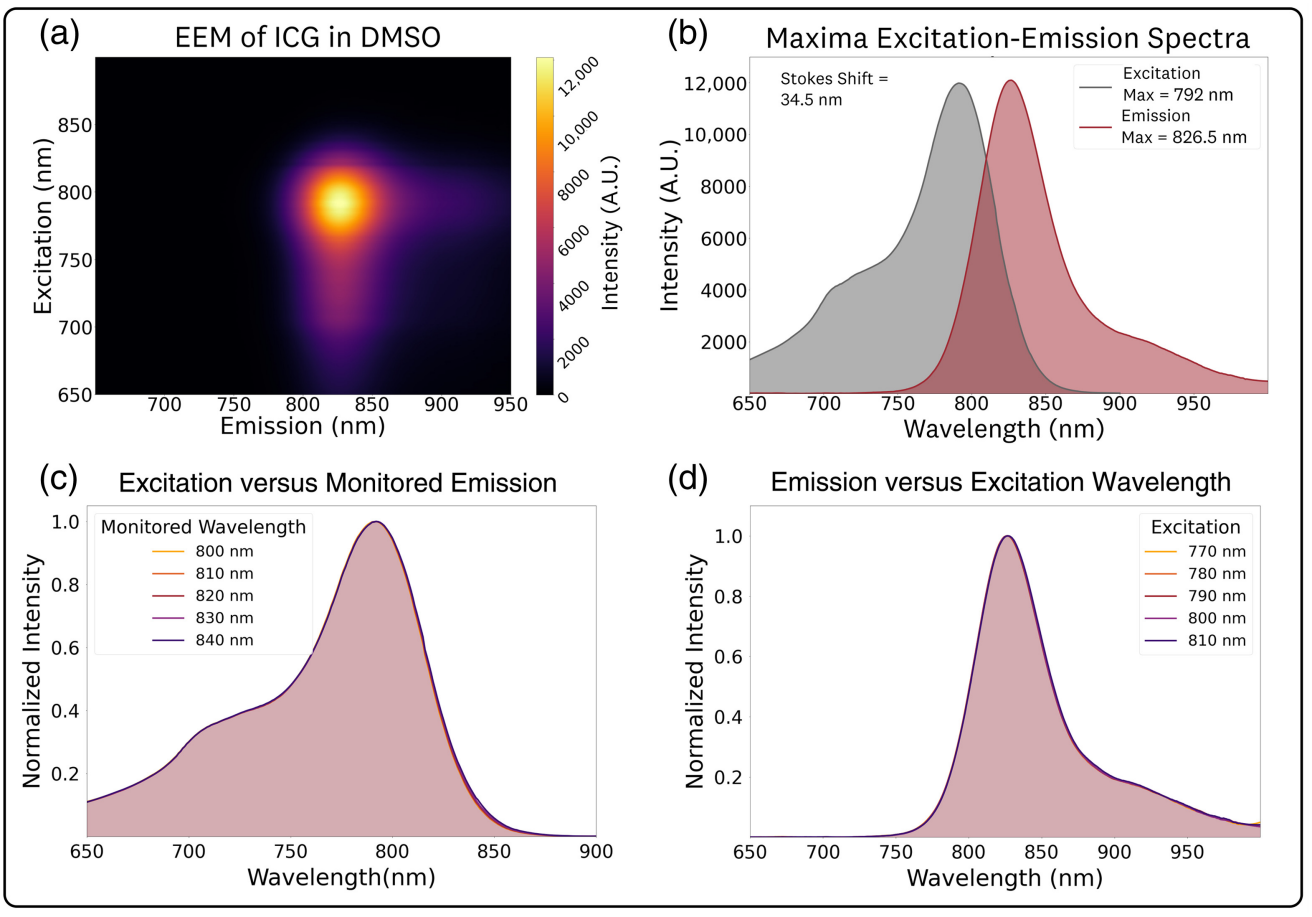
EEM and associated spectra for 1  μM ICG in DMSO: (a) top-down plot of the acquired EEM. (b) Excitation and emission spectra extracted from the EEM maxima. (c) Normalized excitation spectra at varying monitoring emission wavelengths in the 800 to 840 nm range at 10 nm steps. (d) Normalized emission spectra at varying excitation wavelengths in the 770 to 810 nm range at 10 nm steps.

The acquired EEM for 1  μM ICG in DMSO and the associated spectra are shown in Fig. 3. The EEM [Fig. 3(a)] maxima for excitation and emission were measured as 792 and 826.5 nm, respectively. The corresponding excitation and emission spectra at these maxima are plotted in Fig. 3(b), showing a measured Stokes shift of 34.5 nm. The absorbance maximum was measured at 792 nm with a corresponding OD of 0.168.

To assess potential spectral shifts in the excitation, spectra corresponding to monitored emission wavelengths between 800 and 840 nm (10 nm steps) were extracted from the EEM data [Fig. 3(c)]. The identical spectral shapes of the excitation spectra indicate there are no significant shifts in the fluorescence spectral characteristics for varying monitored emission wavelengths.

To evaluate excitation-dependent, spectra from excitation wavelengths in the 770 to 810 nm range (10 nm steps) were extracted from the EEM data [Fig. 3(d)]. The identical spectral shapes of the fluorescence emission indicate there are no significant shifts in the fluorescence spectral characteristics for varying excitation wavelengths. The measured emissions maxima, relative peak intensity, centroid, and relative integrated fluorescence AUC for the various excitations are provided in Table 1. No excitation-dependent shifts were observed in either the emission peak or centroid, confirming that ICG in DMSO follows classical Kasha–Vavilov behavior.

**Table 1:** Emission maxima, relative peak intensities, emission centroids, and relative integrated emission AUC extracted from the EEM of 1  μM ICG in DMSO at excitation wavelengths of 770 to 810 nm (10 nm steps).

Excitation (nm)	Emission peak (nm)	Peak intensity	Emission centroid (nm)	AUC
770	826.5	0.73	836.5	0.73
780	826.5	0.91	836.5	0.91
790	826.5	1.00	836.5	1.00
800	826.5	0.95	836.5	0.95
810	827.0	0.74	837.0	0.75

**Fig. 4:**
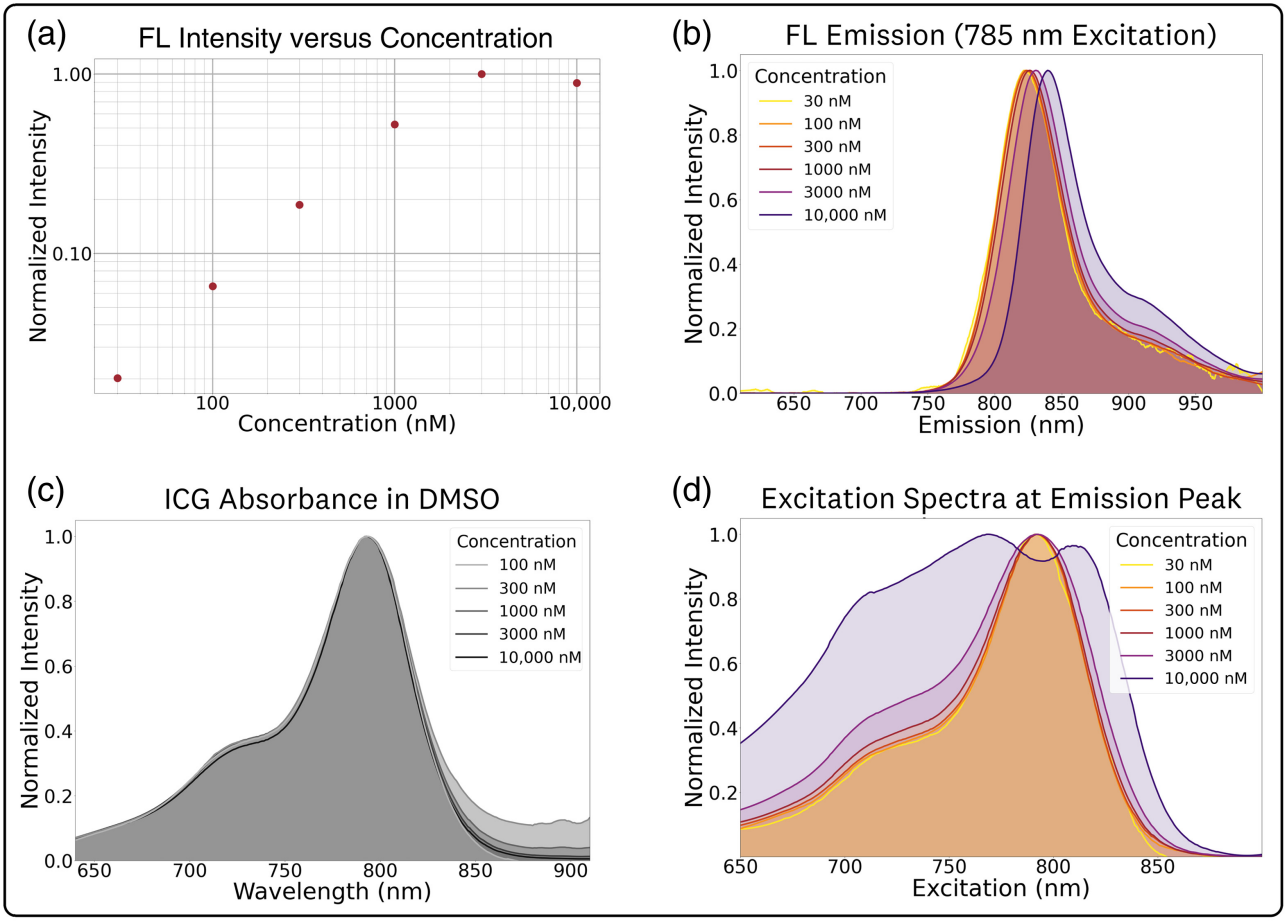
Concentration-dependent spectral effects for ICG in DMSO. (a) Plot of maximum fluorescence intensity versus concentration showing a consistent increase for the 0.03 to 3  μM range with a drop-off in intensity for the 10  μM concentration. (b) Normalized fluorescence emission spectra at 785 nm excitation showing concentration-dependent red shifts. (c) Absorbance spectra show consistent spectral features over the entire concentration while the (d) excitation spectra show broadening and changes due to fluorescence re-absorption for the 3 and 10  μM concentrations.

The acquired EEMs of ICG in DMSO at varying concentrations of 0.03, 0.1, 0.3, 1.0, 3.0, and 10  μM are provided in Fig. S1 of the Supplementary Material. These measurements showed fluorescence quenching at the 10  μM concentration [Fig. 4(a)], concentration-dependent red shifts (CDRS) [Fig. 4(b)], and disparities between absorbance and emission spectra due to inner filter effects (IFE) [Figs. 4(c) and 4(d)]. The log-log plot of maximum fluorescence intensity versus concentration [Fig. 4(a)] shows a constant increase in fluorescence emission versus concentration for the 0.03 to 3  μM range with a drop in intensity for the 10  μM concentration. This drop in fluorescence intensity at the highest concentration is most likely attributed to both primary and secondary inner filter effects (IFE), which correspond to quenching caused by attenuation of the excitation beam and re-absorption of emitted fluorescence, respectively.^67^

To simplify discussion of concentration dependent fluorescence shifts, normalized fluorescence emission spectra at 785 nm excitation were extracted from the EEMs [Fig. 4(b)]. The corresponding measured maximum emission wavelengths and normalized intensities for these plots were (0.03  μM, 822.0 nm, 0.020), (0.1  μM, 823.0 nm, 0.066), (0.3  μM, 824.0 nm, 0.19), (1  μM, 826.0 nm, 0.52), (3  μM, 830.5 nm, 1.0), and (10  μM, 839.5 nm, 0.89). This red-shift in fluorescence emission maxima for increasing concentrations (termed CDRS), primarily results from the re-absorption of emitted fluorescence.^67^

The measured absorbance data are plotted in Fig. 4(c), showing equivalent spectra from the full 0.03 to 10  μM concentration range. Data from the 0.03  μM sample was excluded from Fig. 4(c) due to the low signal-to-noise ratio of its absorbance acquisition. Table S2 in the Supplementary Material contains summarized OD absorbance measurements for the varying concentrations, showing no significant variation in calculated molar extinction coefficients [Table S3 in the Supplementary Material] for the 0.1 to 10  μM range. In contrast to absorbance, the excitation spectra [Fig. 4(d)] at varying concentrations, generated from EEMs at the Fig. 4(b) emission peaks, shows broadening and quenching for the 3 and 10  μM concentrations. This discrepancy between the absorbance and excitation spectra is caused by secondary IFE effects from the re-absorption of fluorescence emission at these high concentrations.^67^

### 3.2 ICG in BSA Solution

**Fig. 5:**
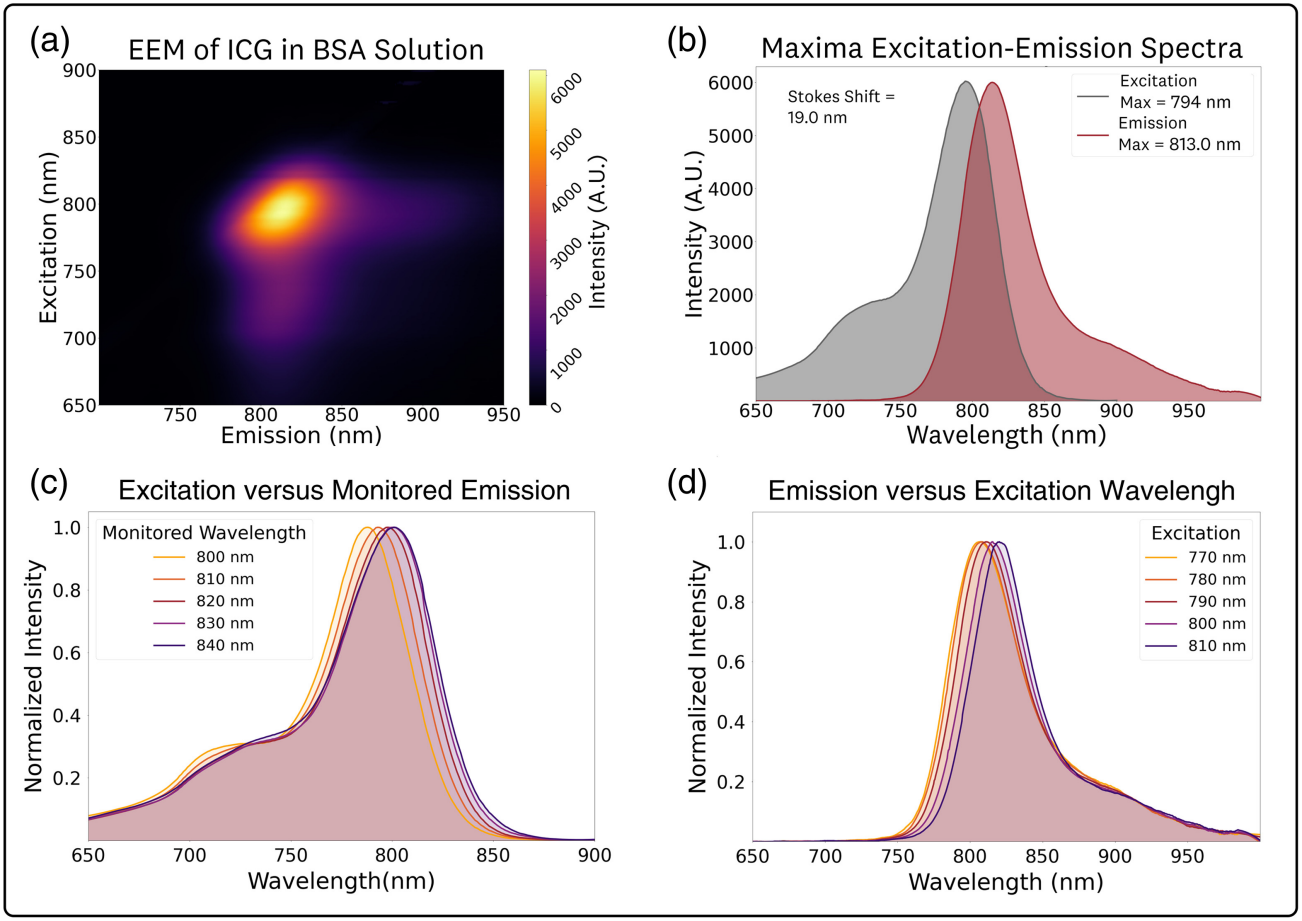
EEM and associated spectra for 1  μM ICG in BSA solution (44  mg/mL). (a) Top-down plot of the acquired EEM showing a “rotation” of the central spectral feature indicating excitation-dependent spectral shifts. (b) Excitation and emission spectra extracted from the EEM maxima. (c) Normalized excitation spectra at varying monitoring emission wavelengths in the 800 to 840 nm range at 10 nm steps. (d) Normalized emission spectra at varying excitation wavelengths in the 770 to 810 nm range at 10 nm steps.

The acquired EEM for 1  μM ICG in BSA solution (44  mg/mL) and associated spectra are shown in Fig. 5. Compared with the ICG in DMSO EEM [Fig. 3(a)], the ICG in BSA solution EEM [Fig. 5(a)] showed a significant “rotation” of the central spectra feature, indicating an excitation-dependent REES and, consequently, a departure from Kasha’s rule, with substantial shifts in the emission spectral characteristics across excitation wavelengths. The EEM maxima for excitation and emission were measured as 794.0 and 813.5 nm, respectively. The corresponding excitation and emission spectra at these maxima are plotted in Fig. 5(b), showing a measured Stokes shift of 19.5 nm. The absorbance maximum was measured at 795 nm with a corresponding OD of 0.142.

To assess spectral shifts in excitation, spectra corresponding to monitored emission wavelengths between 800 and 840 nm (10 nm steps) were extracted from the EEM data [Fig. 5(c)]. The resulting spectra showed increasing shifts in excitation maxima, with measured peaks at 788, 793, 798, 801, and 801 nm for monitored emission wavelengths of 800, 810, 820, 830, and 840 nm, respectively.

To quantify spectral shifts in the fluorescence emission and intensity, spectra from excitation wavelengths in the 760 to 820 nm range (10 nm steps) were extracted from the EEM data [Fig. 5(d)]. The measured emissions maxima, relative peak intensities, centroids, and relative AUC values for the various excitations are summarized in Table 2. The resulting fluorescence emission spectra showed excitation-dependent changes in the peak wavelength and centroid, with only minor changes in photon distribution (spectral broadening) as indicated by the close agreement between peak intensity and AUC ratios. Over the 770 to 810 nm excitation range, the emission peak and centroid exhibited average red-shifts of 0.35 and 0.26 nm per nm of excitation, respectively.

**Table 2:** Emission maxima, relative peak intensities, emission centroids, and relative integrated emission AUC extracted from the EEM of 1  μM ICG in BSA solution (44  mg/mL) at excitation wavelengths of 770 to 810 nm (10 nm steps).

Excitation (nm)	Emission peak	Peak intensity	Emission centroid (nm)	AUC
770	806.5	0.62	819.5	0.66
780	808.0	0.84	820.0	0.88
790	811.0	0.99	822.5	1
800	815.5	1.00	826.0	0.96
810	820.5	0.82	829.5	0.76

**Fig. 6:**
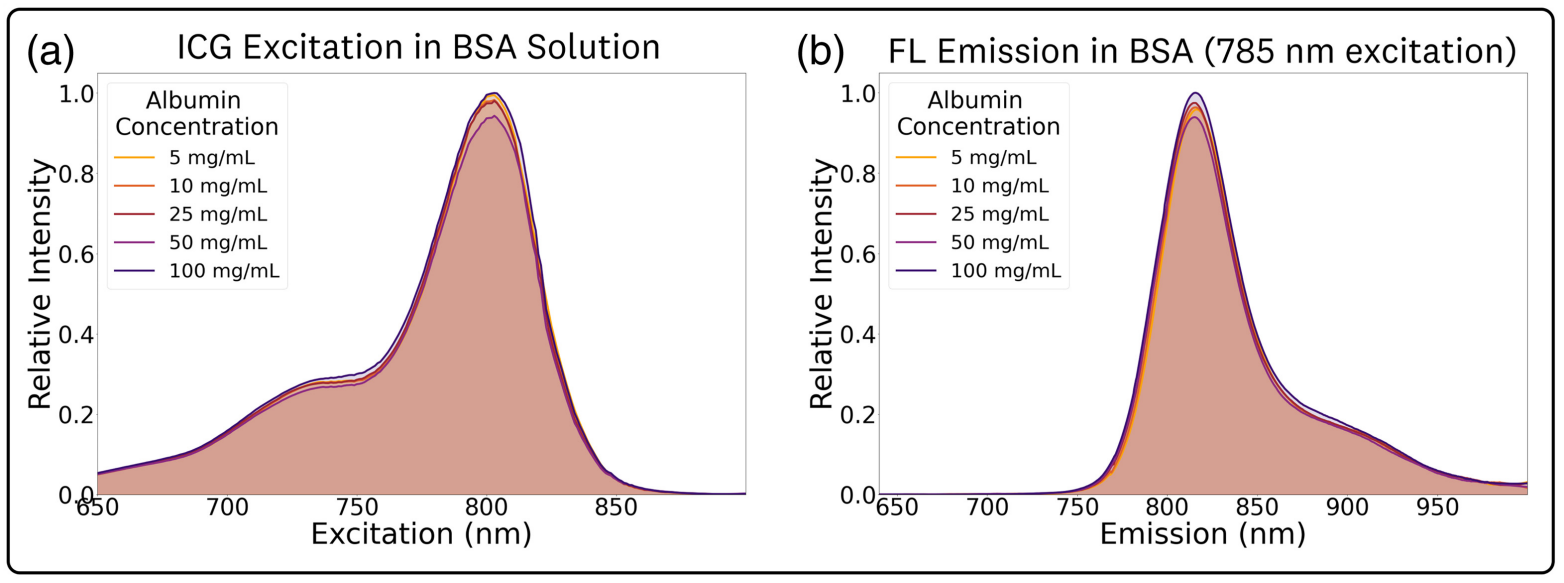
Fluorescence measurements of ICG in 5 to 100  mg/mL BSA solutions at 785 nm excitation: (a) relative excitation spectra obtained at the monitored peak emission wavelength and (b) relative emission spectra, both indicating no significant changes in spectral characteristics across concentrations.

The acquired ICG EEMs at different concentrations of BSA in the 5 to 100  mg/mL range are provided in Fig. S2 of the Supplementary Material. The EEM measurements showed no significant changes in spectral characteristics for varying BSA mg/mL concentrations within the tested range, which covers the full range of biologically relevant concentrations.^59^^,^^60^ To simplify discussion of BSA concentration dependent fluorescence shifts, normalized fluorescence emission spectra at 785 nm excitation were extracted from the EEMs (Fig. 6). The excitation plots [Fig. 6(a)], obtained by monitoring fluorescence at the emission maximum and normalized by the set maxima, show no significant spectral changes, with all excitation peak maxima at 803 nm. Similarly, the fluorescence emission plots [Fig. 6(b)], normalized by the set maxima, show no significant changes in the photon distributions of the emission. The measured maximum emissions and normalized intensities for the various concentrations of BSA were (5  mg/mL, 816.0 nm, 0.96), (10  mg/mL, 815.5 nm, 0.96), (25  mg/mL, 815.5 nm, 0.97), (50  mg/mL, 815.0 nm, 0.94), and (100  mg/mL, 815.5 nm, 1.0).

### 3.3 ICG in 3DP Resin

**Fig. 7:**
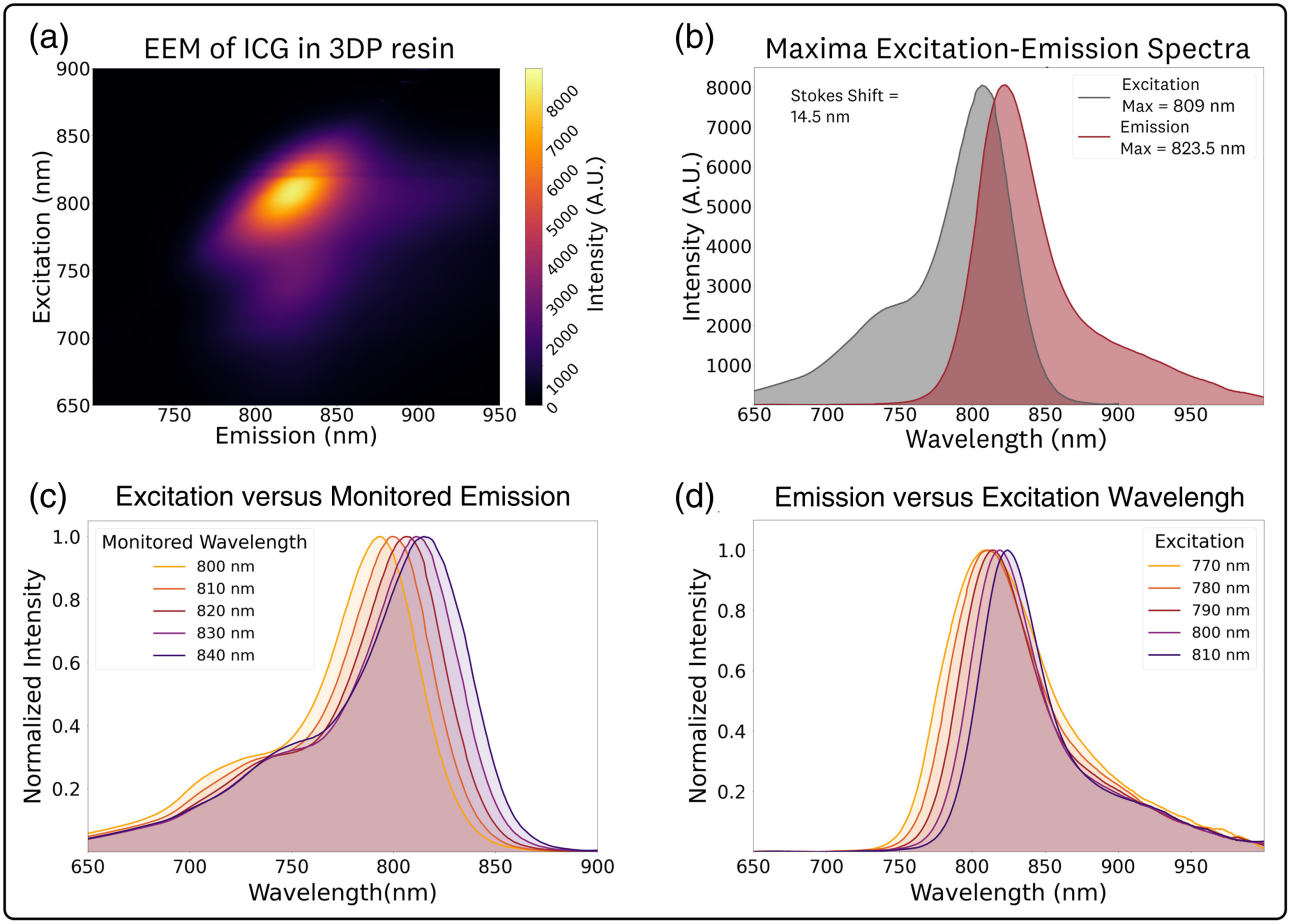
EEM and associated spectra for 1  μM ICG in 3DP resin. (a) Top-down plot of the acquired EEM showing a “rotation” of the central spectral feature indicating excitation-dependent spectral shifts. (b) Excitation and emission spectra extracted from the EEM maxima. (c) Normalized excitation spectra at varying monitoring emission wavelengths in the 800 to 840 nm range at 10 nm steps. (d) Normalized emission spectra at varying excitation wavelengths in the 770 to 810 nm range at 10 nm steps.

The acquired EEM for 1  μM ICG in 3DP resin and associated spectra are shown in Fig. 7. Compared with the ICG in DMSO EEM [Fig. 3(a)], the ICG in 3DP resin EEM [Fig. 7(a)] showed a significant “rotation” of the central spectra feature, indicating an excitation-dependent REES and, consequently, a departure from Kasha’s rule, with substantial shifts in the emission spectral characteristics across excitation wavelengths.^12^ The EEM maxima for excitation and emission were measured as 809 and 823.5 nm, respectively. The corresponding excitation and emission spectra at these maxima are plotted in Fig. 7(b), showing a measured Stokes shift of 14.5 nm. The absorbance maximum was measured at 807 nm with a corresponding OD of 0.159.

To assess spectral shifts in excitation, spectra corresponding to monitored emission wavelengths between 800 and 840 nm (10 nm steps) were extracted from the EEM data [Fig. 5(c)]. The resulting spectra showed increasing shifts in excitation maxima, with measured peaks at 793, 799, 806, 811, and 815 nm for monitored emission wavelengths of 800, 810, 820, 830, and 840 nm, respectively.

**Table 3:** Emission maxima, relative peak intensities, emission centroids, and relative integrated emission AUC extracted from the EEM of 1  μM ICG in 3DP resin at excitation wavelengths of 770 to 810 nm (10 nm steps).

Excitation (nm)	Emission peak (nm)	Peak intensity	Emission centroid (nm)	AUC
770	809.0	0.44	824.5	0.62
780	810.5	0.61	825.0	0.76
790	815.0	0.79	827.5	0.9
800	819.0	0.96	831.0	1
810	824.0	1.00	835.5	0.99

To quantify spectral shifts in the fluorescence emission, spectra from excitation wavelengths in the 760 to 820 nm range (10 nm steps) were extracted from the EEM data [Fig. 7(d)]. The measured emissions maxima, relative peak intensities, centroids, and relative AUC values for the various excitations are summarized in Table 3. The resulting fluorescence emission spectra showed excitation-dependent changes in the peak wavelength and centroid, with noticeable changes in photon distribution (spectral broadening), as reflected by differences between peak intensity and AUC ratios. Over the 770 to 810 nm excitation range, the emission peak and centroid exhibited average red-shifts of 0.39 and 0.26 nm per nm of excitation, respectively.

### 3.4 ICG Spectral Comparison

**Fig. 8:**
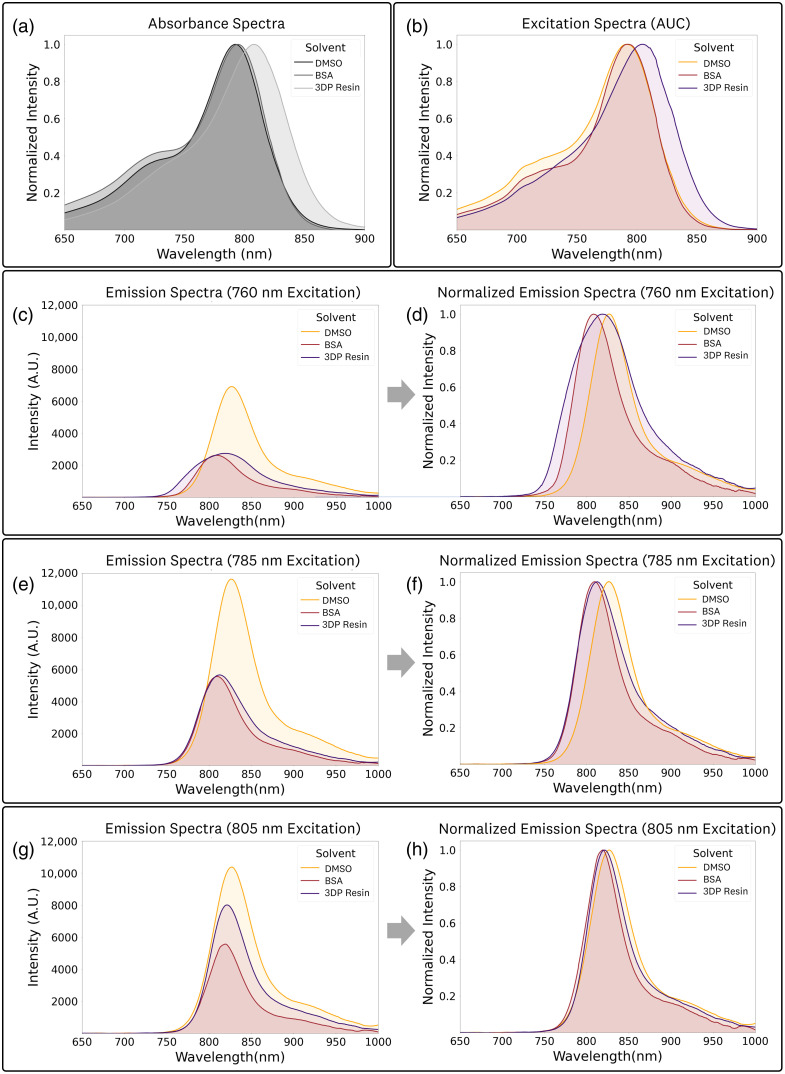
Spectral comparisons of 1  μM ICG in DMSO, BSA solution (44  mg/mL), and 3DP resin: (a) absorbance spectra; (b) excitation spectra calculated from the EEM emission AUC; (c), (d) fluorescence emission spectra at 760 nm excitation; (e), (f) fluorescence emission spectra at 785 nm excitation; (g), (h) fluorescence emission spectra at 805 nm excitation.

The absorbance spectra, excitation spectra for the integrated emission AUC, and emission spectra at 760, 785, and 805 nm excitation for 1  μM ICG in DMSO, BSA solution (44  mg/mL), and 3DP resin are shown in Fig. 8, with summarized data provided in Table 4. Because the BSA solution and 3DP resin exhibited excitation-dependent emission behavior (Figs. 5 and 7), excitation-specific spectra provide an appropriate basis for comparing fluorescence responses across microenvironments in fluorescence sensing applications. Accordingly, fluorescence emission spectra for 760, 785, and 805 nm excitation are shown in Figs. 8(c)–8(h), representing three of the most common excitation wavelengths used for ICG imaging in FGS devices.^7^

The absorbance spectra [Fig. 8(a)] showed maxima at 792, 795, and 807 nm for DMSO, BSA solution, and 3DP resin, respectively. The corresponding peak ODs differed by less than 5% in transmission, calculated as $10^{\text{-}\mathrm{OD}}$ (Table 1). The excitation spectra [Fig. 8(b)], calculated from the integrated emission AUC, showed similar differences in peak positions, with maxima at 791, 792, and 805 nm.

The emission spectra demonstrated notable differences in spectral overlap arising from the excitation-dependent behavior of the BSA solution and 3DP resin samples [Figs. 8(d), 8(f), and 8(h)]. At 760 nm excitation [Figs. 8(c) and 8(d)], the 3DP resin spectra was broad, overlapping with both the DMSO and BSA solution, whereas DMSO and BSA spectra showed limited overlap [Fig. 8(d)]. At 785 nm excitation [Figs. 8(e) and 8(f)], the spectra of the BSA solution and 3DP showed substantial overlap [Fig. 8(f)] with nearly identical AUC intensities [Fig. 8(e), Table 4]. At 805 nm excitation [Figs. 8(g) and 8(h)], the emission spectra of all three samples showed substantial overlap [Fig. 8(h)]. Furthermore, the excitation-dependent peak maxima, centroids, and integrated emission AUC, and photon distributions highlight the need to consider the excitation wavelength and emission collection of the fluorescence sensing device to provide relevant comparisons within varying fluorophore microenvironments and system design parameters. The observed excitation-dependent emissions, leading to nonlinear shifts in the emission peaks and centroids, are further examined in Sec. 3.5.

**Table 4:** Summarized data for the comparison of the spectral behavior of ICG in DMSO, BSA solution, and 3DP resin microenvironments. The integrated emission AUC is normalized to the counts of the DMSO solution emission maxima at the 785 nm excitation.

	Absorbance		Excitation	Emission @760 nm excitation			Emission @785 nm excitation			Emission @805 nm excitation		
	Peak (nm)	OD	Peak (nm)	Peak (nm)	Centroid (nm)	AUC (norm)	Peak (nm)	Centroid (nm)	AUC (norm)	Peak (nm)	Centroid (nm)	AUC (norm)
DMSO	792	0.168	791	826.5	836.5	0.60	826.5	836.5	1.00	827.0	837.0	0.89
Albumin	795	0.142	792	808.0	821.0	0.24	810.0	821.0	0.47	819.5	828.0	0.43
3DP resin	807	0.159	805	819.0	826.5	0.33	813.0	826.0	0.54	821.0	833.0	0.66

### 3.5 Excitation-Dependency and Red-Edge Shift Comparisons

**Fig. 9:**
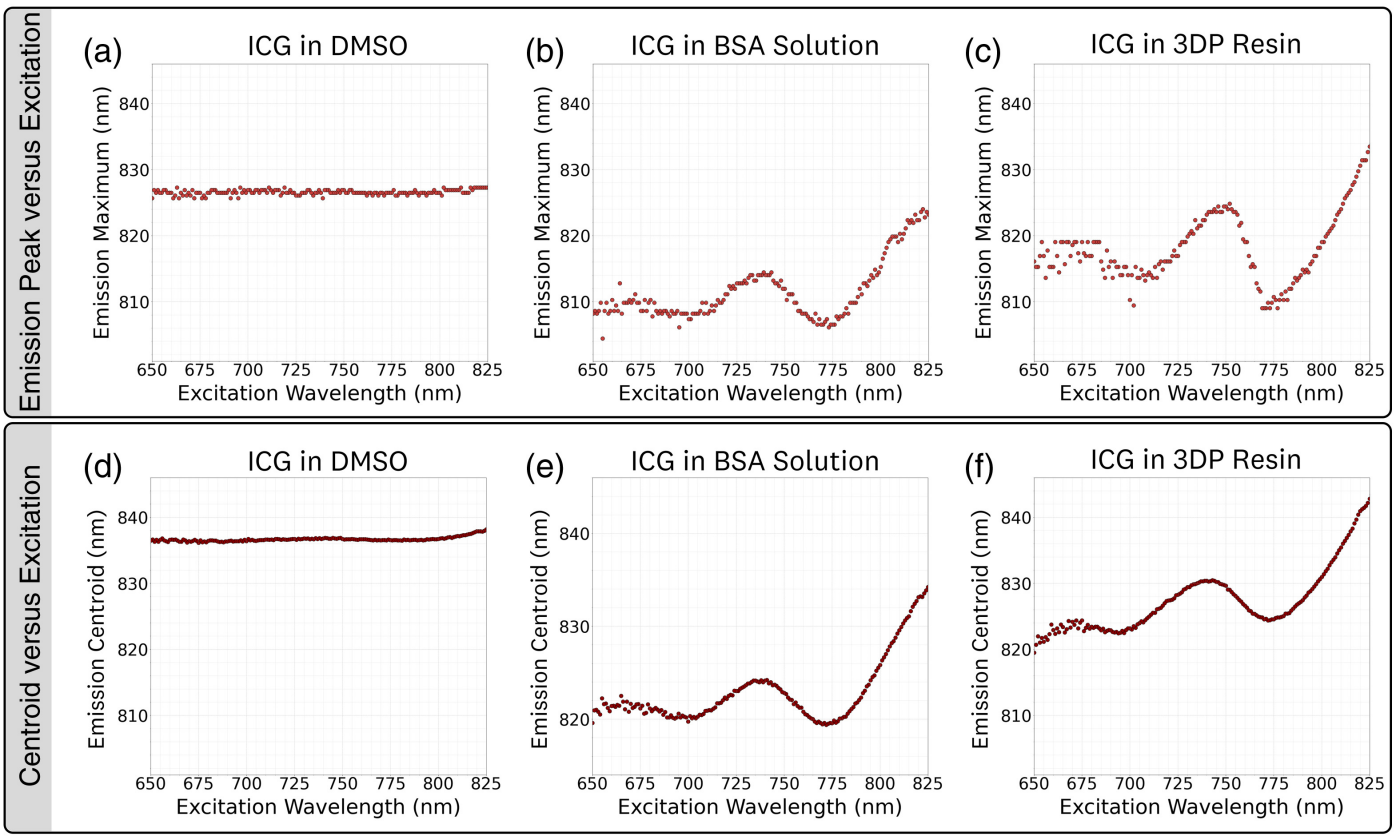
Emission peak (a-c) and centroid (d-f) versus excitation wavelength plot of 1  μM ICG in DMSO, BSA solution, and 3DP resin: The peak emission wavelength for (a) ICG in DMSO showed no significant variation with excitation wavelength while the (b) BSA solution and (c) 3DP resin data showed significant, nonlinear, variations that include REES. The emission centroid wavelength for (d) ICG in DMSO showed no significant variation with excitation wavelength, whereas the (e) BSA solution and (f) 3DP resin data showed significant, nonlinear, variations that include REES.

To further explore excitation-dependent effects on the emission spectra, plots of emission peak versus excitation and emission centroid versus excitation were generated from the respective EEMs for 1  μM ICG in DMSO, BSA solution (44  mg/mL), and 3DP resin (Fig. 9). It is worth noting that the centroid plots showed reduced noise compared with the peak plots due to the variance-reducing effect of AUC integration. For ICG in DMSO [Figs. 9(a) and 9(d)], neither the emission peak nor the centroid wavelength exhibited significant variation with excitation wavelength, consistent with classical Kasha rule behavior and excitation-independent emission. By contrast, ICG in BSA solution [Figs. 9(b) and 9(e)] and in 3DP resin [Figs. 9(c) and 9(f)] showed pronounced shifts in both peak and centroid emission wavelengths as a function of excitation, demonstrating strong nonlinear excitation-dependence and red-edge effects. Both the BSA solution and 3DP resin show a local maxima in the ∼740 to 750 nm range, followed by a pronounced REES trend at excitation wavelengths>770  nm. Notably, this localized increase extends beyond the traditional REES behavior, providing strong evidence of anti-Kasha emission dynamics and suggesting that ICG may undergo multiple relaxation pathways depending on excitation wavelength for the BSA solution and 3DP resin microenvironments.

## 4 Discussion

Here, we reported EEM and absorbance measurements of ICG in DMSO, BSA solutions, and 3DP resin, together with extracted excitation and emission spectra and quantitative summaries of emission peaks, centroids, and integrated emission AUCs. ICG in DMSO exhibited excitation-independent emission consistent with Kasha–Vavilov behavior, whereas the BSA solution and 3DP resin showed significant excitation-dependent emission, including REES and nonlinear departures from Kasha’s rule. To our knowledge, this is the first report of such excitation-dependent phenomena for ICG and for any FGS fluorophore, highlighting the importance of considering excitation-dependent effects in both comparative spectral analysis and sensing system design. The subsequent subsections provide detailed discussion of each set of results.

### 4.1 ICG in DMSO

The EEM for 1  μM ICG in DMSO (Fig. 3) provided ‘symmetric’ spectra throughout the varying excitation wavelengths with measured emission peaks of 826.5±0.5  nm over the 760 to 810 nm excitation range. This EEM confirms the conventional Kasha-rule fluorophore behavior that assumes invariant spectral photon distribution of fluorescence emission with excitation wavelength.

The EEMs and absorbance at varying ICG concentrations in DMSO (Fig. 4) show the effects of concentration-dependent fluorescence shifts including fluorescence quenching, CDRS, and disparities between the absorbance and excitation spectra due to IFEs. Increases in fluorophore concentration lead to a proportional increase in fluorescence emission intensity within a linear range, followed by a decrease at higher concentrations due to IFEs or fluorophore aggregation; this concentration-quenching is well understood and reported both experimentally and through Monte Carlo photon transport simulations.^67^^–^^69^ The measured linear range for ICG in DMSO was 0.3 to 3  μM with the 10  μM sample showing significant quenching, emission shifts (CDRS) and distortion of the excitation spectra. The 3  μM sample exhibited minor quenching, CDRS, and excitation spectra broadening. Within the linear range, the measured EEMs (Fig. S1 in the Supplementary Material) demonstrated no significant differences, displaying an anticipated increase in emission intensity proportional to concentration across the entire wavelength matrix. The spectral distribution of the absorbance spectra was consistent along the full concentration range, which indicates that the observed fluorescence shifts are due to IFEs and not fluorophore aggregation at these high concentrations. The photon distribution of the absorbance and excitation spectra were equivalent within the identified linear range. It is worth noting that this measured linearity range is specific to the geometry of the spectral measurement, which uses an orthogonal excitation-emission collection within a 10 mm pathlength cuvette. These results highlight the need to understand the linearity range of a fluorophore to adequately choose concentrations for EEM and fluorescence spectra characterization and confirm that concentration-dependent effects (quenching, CDRS, and exciation-spectra distortion arising from IFEs) are mechanistically and spectrally distinct from the excitation-dependent emission behavior observed in the BSA solution and 3DP resin microenvironments.

### 4.2 ICG in BSA Solution

In contrast to the ICG in DMSO sample, the 1  μM ICG in BSA solution (Fig. 5) showed a “rotation” of the central spectral feature indicating excitation-dependent emission, REES, and departures from Kasha’s rule. This nonlinear effect is most likely attributed to albumin binding^21^^,^^70^ causing changes in solvent relaxation times, where the excitation red shift is observed because relaxation is not complete before fluorescence emission occurs.^12^ Although steady-state EEM measurements do not resolve individual microscopic binding configurations of ICG, the smooth continuous excitation-dependent shift observed here is consistent with REES arising from a distribution of local relaxation environments and is distinct from the spectral superposition expected for discrete ICG populations. If the observed behavior arose from discrete ICG populations (e.g., monomeric, H-aggregated, or J-aggregated states), the EEM would be expected to reflect a superposition of fixed spectral components, such as multiple ridges, shoulders, or excitation-dependent reweighting of stationary bands. The smooth continuous shift or “rotation” observed here does not match that behavior and indicates that the excitation-dependent emission cannot be explained by such mixed-state populations.^31^^,^^71^^–^^73^ Quantitatively, average emission red-shift slopes of $0.35\;\mathrm{nm}\cdot {\mathrm{nm}}^{-1}$ (peak) and $0.26\;\mathrm{nm}\cdot {\mathrm{nm}}^{-1}$ (centroid) were observed over the 770 to 810 nm excitation range (Table 2), with only minor changes in spectral photon distributions. These results emphasize the importance of EEM fluorophore measurements to characterize excitation-dependent effects associated with biologically relevant microenvironments.

EEM measurements for ICG in varying BSA concentration solutions [Fig. 6, Fig. S2 in the Supplementary Material) showed the same excitation-dependent behavior, with no significant spectral differences within the biologically relevant albumin concentrations in the 5 to 100  mg/mL range.^59^^,^^60^ As an additional confirmation, supplementary absorbance and EEM measurements for 100 and 1000 nM ICG in 10  mg/mL BSA (Fig. S3 in the Supplementary Material) showed the same excitation-dependent behavior despite the tenfold difference in ICG concentration, indicating that the observed effect is not driven by absorption-dependent attenuation or concentration-dependent effects. Consideration of excitation-dependent emission shifts for ICG in BSA solutions could help optimize fluorescence sensing designs including FGS imagers given the relevancy to perfusion and *in vivo* applications. The measured ICG EEM in the BSA solution (Fig. 5) provides spectral information for albumin concentrations found in whole blood,^60^^,^^74^ thus serving as a good estimator of *in vivo* ICG spectra for perfusion applications. Furthermore, the invariability of the EEM within the wide albumin concentration range indicates that it might be a suitable estimator of interstitial ICG spectra.^74^

It is worth noting that while higher BSA concentrations introduced greater excitation scattering peaks in the raw data, these effects were successfully addressed by the robust smoothing approach (see Sec. 2.6), yielding equivalent EEMs and emission spectra across the full concentration range (Fig. 6, Fig. S2 in the Supplementary Material). These results indicate that bulk turbidity/scattering of the solution is not responsible for the excitation-dependent effects observed for ICG in BSA. Rather, it demonstrates that the smoothing technique can accommodate varying levels of scattering without negatively affecting the processed results or introducing artifacts within the tested signal-to-noise ratios and smoothing span.

### 4.3 ICG in 3DP Resin

Similar to the ICG in BSA solution EEM, the ICG in 3DP resin EEM (Fig. 7) showed a “rotation” of the central spectral feature indicating excitation-dependent emission, REES, and departures from Kasha’s rule. Quantitatively, the average emission red-shift slopes were $0.39\;\mathrm{nm}\cdot {\mathrm{nm}}^{-1}$ (peak) and $0.26\;\mathrm{nm}\cdot {\mathrm{nm}}^{-1}$ (centroid) over 770 to 810 nm, with noticeable changes to spectral photon distribution (broadened spectra) evident from differences between peak-intensity and AUC trends (Table 3). These nonlinear effects are most likely attributed to the embedding of the fluorophore within a solid polymer matrix, causing changes in relaxation times, where the excitation red shift is observed because relaxation is not complete before fluorescence emission occurs.^12^ Given the intended use of these 3DP fluorescent materials as “ground-truth” measurements, in the form of reference targets and phantoms,^7^^,^^22^^,^^23^^,^^57^ the presence of excitation-dependent emission underscores the need to consider the excitation wavelength for accurate, application-specific, and system-specific characterization and comparisons. The role of the 3DP material in the present study is therefore not to replicate the full optical transport conditions of *in vivo* tissue imaging, but to establish its spectral behavior as a fluorescent reference material for phantom development, benchmarking, and simulation. In this context, the excitation-dependent behavior and spectral overlap with albumin-bound ICG are directly relevant to the use of these materials in tissue-mimicking phantom studies and in experimentally validated fluorescence Monte Carlo and digital-twin workflows.^7^^,^^22^^,^^23^^,^^57^^,^^69^ When used for tissue-mimicking phantom manufacturing, the 3DP resin is generally further tuned with absorbing chromophores and scattering agents to achieve tissue-relevant absorption and reduced scattering coefficients. This additional optical-property tuning was outside the scope of the present spectroscopic study but has been demonstrated in related work from our group.^22^^,^^57^^,^^69^

### 4.4 ICG Spectral Comparison

The spectral comparison of 1  μM ICG in DMSO, BSA solution, and 3DP resin (Fig. 8), showed shifts in spectral emission wavelengths and distributions, including excitation-dependent effects in the BSA and 3DP samples. Given the excitation-dependent spectral behavior, and because fluorescence sensing relies on emitted light, emission spectra at defined excitation wavelengths provide the most appropriate basis for comparison. In this context, the BSA solution and 3DP resin samples exhibited substantial spectral overlap at excitation wavelengths commonly used in FGS devices (760 to 805 nm, Figs.  8(d), 8(f), and 8(h)], with cosine similarities of 0.891 and 0.994 at 760 and 785 nm excitation, respectively, as calculated from the normalized dot product of the emission spectral vectors.^75^^,^^76^ This spectral overlap indicates that ICG in 3DP resin can serve as an adequate surrogate reference material for albumin-bound ICG under clinically relevant excitation illumination, whereas the degree of equivalence between the two microenvironments is excitation-dependent (Figs. 8 and 9). Accordingly, comparisons involving fluorescent phantoms or surrogate materials should use excitation-specific spectra rather than assume a single excitation-independent emission profile. Rigorous equivalency assessment further requires accounting for both the excitation conditions and the specific emission collection parameters of the sensing system.

Considering that the fluorophore spectral emission can vary with both microenvironment and excitation wavelength, the most accurate method for comparing fluorescence emission intensity involves integrating the excitation-specific emission spectra within the sensing system’s detection band (such as long-pass emission collection, band-pass emission collection, etc). The acquired EEM dataset facilitates application and system-specific comparisons, incorporating considerations of both excitation and emission collection parameters. It is important to note that the provided EEMs (Supplementary Data Files) are directly comparable in their reported intensities because identical acquisition parameters were used. Although not explored in our analysis, it is worth noting that ‘weighted emission spectra’ can be generated from the provided EEMs for broadband excitation sources.

### 4.5 Excitation-Dependency and Red-Edge Shift Comparisons

The peak and centroid versus excitation plots (Fig. 9) showed three distinct behaviors. In DMSO, emission is effectively excitation-independent, following Kasha’s rule, and serves as the baseline for traditional fluorophore behavior assumptions. In BSA solution and 3DP resin, pronounced red-edge excitation shifts emerge for excitations ≥∼770  nm, indicating that varied excitation alongside partial vibronic relaxation produce excitation-dependent emission. In addition, a localized maxima near 740 to 750 nm precedes the REES trend in both media, pointing to extra microstate selectivity not captured by a simple monotonic shift and representing a more complex departure from Kasha’s rule. The detailed mechanistic explanation for these complex excitation-dependent effects lies beyond the scope of the present work but represents an important direction for further study.

To contextualize the magnitude of these excitation-dependent effects for practical fluorescence sensing systems, we considered a representative FGS detection configuration and used the ICG in BSA EEM data to estimate the emitted signal captured within a typical detection band. For a system using 805 nm excitation with an 815 nm long-pass emission filter,^7^ the integrated emission above the filter cut-on corresponds to ∼40.5% of the total fluorescence AUC for the albumin-bound ICG spectrum. If that same system were modified to use 775 nm excitation to accommodate future multifluorophore imaging in the ∼800  nm range while retaining the 815 nm long-pass filter, an excitation-independent assumption would suggest comparable or slightly improved performance, because the total fluorescence AUC at 775 nm excitation is ∼4.8% higher than at 805 nm. However, when the measured excitation-dependent emission spectrum is considered, the fraction of emission transmitted above 815 nm at 775 nm excitation is ∼30.6% rather than ∼40%, corresponding to an ∼24.5% reduction in detected signal throughput. This example further highlights, for ICG, the need to consider excitation-specific spectra in system design and comparison, because excitation-independent spectral assumptions can lead to incorrect estimates of the fluorescence collected within a defined emission detection band. Similar considerations may also apply to other fluorophores used or currently under development for fluorescence sensing if they exhibit excitation-dependent emission behavior.

### 4.6 Future Work and Limitations

The presented EEM and absorbance measurements provided comprehensive insights into ICG excitation-dependent spectral shifts in varying microenvironments but were limited to room-temperature conditions. It is worth noting that the captured EEMs contain some “banding” along the emission axis [see Figs. 3(a), 5(a), and 7(a)] for excitation wavelengths in the ∼820 to 860 nm range. These artifacts result from limitations in the spectrofluorometer’s correction of xenon lamp spectral peaks during intensity normalization, as confirmed through communication with the manufacturer. Because the robust smoothing applied to the data was performed independently on each emission spectrum rather than across the excitation axis (the y-axis in the top-down EEM plots), these banding artifacts were not removed. However, the reported peak emissions, centroids, and integrated AUCs, as well as the extracted excitation and emission spectra, were minimally affected, because the relevant excitation range for ICG fluorescence sensing applications lies within the 650 to 820 nm range. It is also worth noting that the 3DP sample was measured in a printed cuvette with a nominal 12 mm pathlength, compared with the 10 mm standard cuvettes used for the liquid samples, which may introduce some disparity in inner-filter and scattering contributions; however, because the spectrofluorometer used an orthogonal excitation-emission geometry that primarily probes the central excitation-emission overlap region, the effect of this pathlength difference is limited.

Further insights could be gained by including whole blood in the suite of tested solvents. Although the high absorption and scattering in whole blood may pose measurement challenges, understanding excitation-dependence in this context would improve the *in vivo* spectral estimations. Moreover, conducting *in vivo* fluorescence spectral measurements, which would require specialized equipment, could further characterize the effects of albumin binding on *in vivo* ICG EEM shifts. The present study characterizes the intrinsic excitation–emission behavior established by the local albumin or resin microenvironment; surrounding tissue absorption and scattering primarily modify excitation fluence and emission propagation, including preferential attenuation of shorter-wavelength emission, rather than the underlying fluorophore–matrix photophysics.^77^ This separation has been supported in related experimentally validated fluorescence Monte Carlo and digital-twin studies from our group, which reproduced fluorescence behavior across tissue-relevant variations in absorption, scattering, fluorophore concentration, depth, and geometry, although direct spectral confirmation of the excitation-dependent shifts under tissue-like conditions remains an area for future study.^22^^,^^57^^,^^69^

Characterizing how temperature influences EEMs and excitation-dependent behavior would provide valuable insights. In addition, IFE corrections^78^ and spectral unmixing of EEMs could further help advance fluorophore characterization, including understanding of nonlinear and aggregation effects. Future studies that measure absolute quantum yields at varying excitation wavelengths in BSA solutions and 3DP resin could provide further insight into the extent of deviation from Kasha–Vavilov behavior.

As FGS-targeted fluorophores continue to develop, measuring EEM spectra for bound fluorophores that mimic the *in vivo* environment will be crucial for understanding their behavior and the extent of excitation-dependence and deviations from Kasha’s rule. These assessments are further complicated by the diverse chemical environments of targeted binding sites. If solvent-related excitation effects are found to be minimal, this could indicate “robust” spectral properties, with little to no shift observed despite changes in microenvironment. Conversely, pronounced excitation-dependent spectral changes would be important to identify prior to clinical application, as they could inform imaging system design or motivate modification of the fluorophore structure.

## 5 Conclusion

This study presents comprehensive EEM characterization of ICG in DMSO, BSA solutions, and 3DP resin microenvironments, revealing significant excitation-dependent emission, including REES and nonlinear departures from Kasha’s rule, in the BSA solution and 3DP resin; by contrast, DMSO exhibited traditional excitation-independent (Kasha–Vavilov) behavior. These results provide, to our knowledge, the first documentation of REES and broader deviation from Kasha’s rule for ICG and any FGS-associated fluorophore. The findings highlight the need to account for excitation-dependent emission when comparing spectra across systems and microenvironments. The observed excitation-dependent shifts also support the use of excitation-specific, EEM-derived spectra for system design, intersystem comparisons, and phantom development; notably, the substantial emission overlap between BSA solution and 3DP resin spectra at commonly used excitation wavelengths (∼760 to 805 nm) suggests that ICG in 3DP resin can serve as a stable surrogate reference for albumin-bound ICG when system excitation and emission collection parameters are considered.^7^^,^^22^^,^^57^ The EEM datasets provided here offer a practical reference for fluorophore characterization and benchmarking that incorporate excitation-dependent effects. Future work extending these measurements to additional physiological conditions (i.e., temperature variation, whole blood, and *in vivo* contexts) and absolute quantum-yield assessments at varying excitations will further clarify excitation-dependent behavior and its implications for fluorophore chemistry, spectral comparisons, and fluorescence-based imaging system design.

## Supplementary Material

10.1117/1.JBO.31.7.077003.s01

## Data Availability

Data and code are available upon reasonable request.

## References

[1] Valeur B. Berberan-Santos M. N., “Chemical sensing via fluorescence,” in Molecular Fluorescence, pp. 409–478, John Wiley & Sons, Ltd (2012).

[2] Croce A. C. Bottiroli G., “Autofluorescence and fluorescence labeling in biology and medicine,” in Molecular Fluorescence, pp. 479–505, John Wiley & Sons, Ltd (2012).

[3] Yang Z. et al., “Macro-/micro-environment-sensitive chemosensing and biological imaging,” Chem. Soc. Rev. 43(13), 4563–4601 (2014). 10.1039/C4CS00051J 24723011

[4] Mchedlov-Petrossyan N. O. Vodolazkaya N. A. Doroshenko A. O., “Ionic equilibria of fluorophores in organized solutions: the influence of micellar microenvironment on protolytic and photophysical properties of rhodamine B,” J. Fluoresc. 13, 235–248 (2003). JOFLEN 1053-0509 10.1023/A:1025089916356

[5] Koch M. Symvoulidis P. Ntziachristos V., “Tackling standardization in fluorescence molecular imaging,” Nat. Photonics 12(9), 505–515 (2018). NPAHBY 1749-4885 10.1038/s41566-018-0221-5

[r6] Pogue B. W. et al., “Fluorescence-guided surgery and intervention—an AAPM emerging technology blue paper,” Med. Phys. 45(6), 2681–2688 (2018). MPHYA6 0094-2405 10.1002/mp.12909 29633297 PMC9560243

[r7] Ochoa M. I. et al., “Assessment of open-field fluorescence guided surgery systems: implementing a standardized method for characterization and comparison,” J. Biomed. Opt. 28(9), 096007 (2023). JBOPFO 1083-3668 10.1117/1.JBO.28.9.096007 37745774 PMC10513724

[8] DSouza A. V. et al., “Review of fluorescence guided surgery systems: identification of key performance capabilities beyond indocyanine green imaging,” J. Biomed. Opt. 21(8), 080901 (2016). JBOPFO 1083-3668 10.1117/1.JBO.21.8.080901 27533438 PMC4985715

[9] “Solvent and environmental effects,” in Principles of Fluorescence Spectroscopy, Lakowicz J. R., Ed., pp. 205–235, Springer US, Boston, MA (2006).

[10] Valeur B. Berberan-Santos M. N., “Environmental effects on fluorescence emission,” in Molecular Fluorescence, pp. 109–140, John Wiley & Sons, Ltd (2012). 10.1002/9783527650002.ch5

[11] Chattopadhyay A. Mukherjee S., “Fluorophore environments in membrane-bound probes: a red edge excitation shift study,” Biochemistry 32(14), 3804–3811 (1993). BICHAW 0006-2960 10.1021/bi00065a037 8466919

[r12] Lakowicz J. R., “Dynamics of solvent and spectral relaxation,” in Principles of Fluorescence Spectroscopy, pp. 237–276, Springer US, Boston, MA (2006). 10.1007/978-0-387-46312-4_7

[13] Khara D. C. Samanta A., “Solvation dynamics and red-edge effect of two electrically charged solutes in an imidazolium ionic liquid,” Indian J. Chem. 49(5-6), 714–720 (2010).

[14] Kasha M., “Characterization of electronic transitions in complex molecules,” Discuss. Faraday Soc. 9, 14 (1950). DFSOAW 0014-7664 10.1039/df9500900014

[15] Vavilov S. I., “The dependence of the intensity of the fluorescence of dyes upon the wavelength of the exciting light,” Lond. Edinb. Dublin Philos. Mag. J. Sci. 43(254), 307–320 (1922). 10.1080/14786442208565217

[16] Maafi M., “Kasha’s rule and photochemistry,” in Photokinetics: A New Perspective, Maafi M., Ed., pp. 73–117, Springer Nature Switzerland, Cham (2025).

[r17] Prasad A. K. Jain M., “Breakdown of Kasha’s rule in a ubiquitous, naturally occurring, wide bandgap aluminosilicate (Feldspar),” Sci. Rep. 8(1), 810 (2018). SRCEC3 2045-2322 10.1038/s41598-017-17466-z 29339737 PMC5770446

[r18] Tseng H.-W. et al., “Excited-state intramolecular proton-transfer reaction demonstrating anti-Kasha behavior,” Chem. Sci. 7(1), 655–665 (2016).1478-6524 10.1039/C5SC01945A 29896352 PMC5952995

[r19] Uttamlal M. Holmes-Smith A. S., “The excitation wavelength dependent fluorescence of porphyrins,” Chem. Phys. Lett. 454(4–6), 223–228 (2008). CHPLBC 0009-2614 10.1016/j.cplett.2008.02.012

[20] Veys K. Escudero D., “Anti-Kasha fluorescence in molecular entities: central role of electron–vibrational coupling,” Acc. Chem. Res. 55(18), 2698–2707 (2022). ACHRE4 0001-4842 10.1021/acs.accounts.2c00453 36048561

[21] Yoneya S. et al., “Binding properties of indocyanine green in human blood,” Invest. Ophthalmol. Vis. Sci. 39(7), 1286–1290 (1998). 9620093

[22] Ruiz A. J. et al., “3D printing fluorescent material with tunable optical properties,” Sci. Rep. 11(1), 17135 (2021). SRCEC3 2045-2322 10.1038/s41598-021-96496-0 34429467 PMC8384872

[23] Ruiz A. J. et al., “Indocyanine green matching phantom for fluorescence-guided surgery imaging system characterization and performance assessment,” J. Biomed. Opt. 25(5), 056003 (2020). JBOPFO 1083-3668 10.1117/1.JBO.25.5.056003 32441066 PMC7240319

[24] Zelken J. A. Tufaro A. P., “Current trends and emerging future of indocyanine green usage in surgery and oncology: an update,” Ann. Surg. Oncol. 22(S3), 1271–1283 (2015). 10.1245/s10434-015-4743-5 26193966

[r25] Alander J. T. et al., “A review of indocyanine green fluorescent imaging in surgery,” Int. J. Biomed. Imaging 2012(1), 940585 (2012). 10.1155/2012/940585 22577366 PMC3346977

[26] Dzurinko V. L. Gurwood A. S. Price J. R., “Intravenous and indocyanine green angiography,” Optom. - J. Amer. Optom. Assoc. 75(12), 743–755 (2004). 10.1016/S1529-1839(04)70234-1 15624671

[27] Koch M. Ntziachristos V., “Advancing surgical vision with fluorescence imaging,” Annu. Rev. Med. 67(1), 153–164 (2016). 10.1146/annurev-med-051914-022043 26768238

[28] Marshall M. V. et al., “Near-infrared fluorescence imaging in humans with indocyanine green: a review and update,” Open Surg. Oncol. J. Online 2(2), 12–25 (2010). 10.2174/1876504101002010012 PMC3424734 22924087

[29] Landsman M. L. et al., “Light-absorbing properties, stability, and spectral stabilization of indocyanine green,” J. Appl. Physiol. 40(4), 575–583 (1976). JAPYAA 0021-8987 10.1152/jappl.1976.40.4.575 776922

[r30] Yuan B. Chen N. Zhu Q., “Emission and absorption properties of indocyanine green in Intralipid solution,” J. Biomed. Opt. 9(3), 497–503 (2004). JBOPFO 1083-3668 10.1117/1.1695411 15189087 PMC1533769

[r31] Chon B. et al., “Indocyanine green (ICG) fluorescence is dependent on monomer with planar and twisted structures and inhibited by H-aggregation,” Int. J. Mol. Sci. 24(17), 13030 (2023).1422-0067 10.3390/ijms241713030 37685837 PMC10488082

[r32] Mačianskienė R. et al., “Spectral characteristics of voltage-sensitive indocyanine green fluorescence in the heart,” Sci. Rep. 7(1), 7983 (2017). SRCEC3 2045-2322 10.1038/s41598-017-08168-7 28801595 PMC5554165

[r33] Yu A. et al., “Solvatochromism and solvation dynamics of structurally related cyanine dyes,” J. Phys. Chem. A 106(41), 9407–9419 (2002). JPCAFH 1089-5639 10.1021/jp0205867

[34] Bongsu Jung V. I. V. Anvari B., “Revisiting indocyanine green: effects of serum and physiological temperature on absorption and fluorescence characteristics,” IEEE J. Sel. Top. Quantum Electron. 20(2), 149–157 (2014). IJSQEN 1077-260X 10.1109/JSTQE.2013.2278674

[35] Li Y. et al., “A human serum albumin-indocyanine green complex offers improved tumor identification in fluorescence-guided surgery,” Transl. Cancer Res. 13(1), 437–452 (2024). 10.21037/tcr-23-2338 38410209 PMC10894326

[r36] Cheung C. C. L. et al., “Liposome-templated indocyanine green J- aggregates for, *in vivo*, near infrared imaging and stable photothermal heating,” Nanotheranostics 4(2), 91–106 (2020). 10.7150/ntno.41737 32190536 PMC7064739

[r37] Kraft J. C. Ho R. J. Y., “Interactions of indocyanine green and lipid in enhancing near-infrared fluorescence properties: the basis for near-infrared imaging *in vivo*,” Biochemistry 53(8), 1275–1283 (2014). BICHAW 0006-2960 10.1021/bi500021j 24512123 PMC3985908

[r38] Stern N. B. Shrestha B. Porter T., “A facile approach to producing liposomal J-aggregates of indocyanine green with diagnostic and therapeutic potential,” Adv. Ther. 7(8), 2400042 (2024). 10.1002/adtp.202400042 PMC11308451 39132131

[r39] Cho J. et al., “Monitoring distribution of the therapeutic agent dimethyl sulfoxide via solvatochromic shift of albumin-bound indocyanine green,” Sensors 23(18), 7728 (2023). SNSRES 0746-9462 10.3390/s23187728 37765785 PMC10535274

[r40] Gamage R. S. Smith B. D., “Spontaneous transfer of indocyanine green from liposomes to albumin is inhibited by the antioxidant α-tocopherol,” Langmuir 38(39), 11950–11961 (2022). LANGD5 0743-7463 10.1021/acs.langmuir.2c01715 36126324 PMC9897306

[r41] Berezin M. Y. et al., “Near infrared dyes as lifetime solvatochromic probes for micropolarity measurements of biological systems,” Biophys. J. 93(8), 2892–2899 (2007). BIOJAU 0006-3495 10.1529/biophysj.107.111609 17573433 PMC1989699

[r42] Vinegoni C. et al., “Indocyanine green enables near-infrared fluorescence imaging of lipid-rich, inflamed atherosclerotic plaques,” Sci. Transl. Med. 3(84), 84ra45 (2011). STMCBQ 1946-6234 10.1126/scitranslmed.3001577 PMC3112179 21613624

[r43] Patel R. H. et al., “Multifunctionality of indocyanine green-loaded biodegradable nanoparticles for enhanced optical imaging and hyperthermia intervention of cancer,” J. Biomed. Opt. 17(4), 046003 (2012). JBOPFO 1083-3668 10.1117/1.JBO.17.4.046003 22559681

[44] Altınoǧlu E. I. et al., “Near-infrared emitting fluorophore-doped calcium phosphate nanoparticles for, *in vivo*, imaging of human breast cancer,” ACS Nano 2(10), 2075–2084 (2008). ANCAC3 1936-0851 10.1021/nn800448r 19206454

[45] Starosolski Z. et al., “Indocyanine green fluorescence in second near-infrared (NIR-II) window,” PLOS ONE 12(11), e0187563 (2017). POLNCL 1932-6203 10.1371/journal.pone.0187563 29121078 PMC5679521

[r46] Kochubey V. I., “Spectral characteristics of indocyanine green upon its interaction with biological tissues,” Opt. Spectrosc. 99(4), 560–566 (2005). OPSUA3 0030-400X 10.1134/1.2113369

[r47] Desmettre T. Devoisselle J. M. Mordon S., “Fluorescence properties and metabolic features of indocyanine green (ICG) as related to angiography,” Surv. Ophthalmol. 45(1), 15–27 (2000). 10.1016/S0039-6257(00)00123-5 10946079

[48] Kanniyappan U. et al., “Near-infrared fluorescence image quality test methods for standardized performance evaluation,” Proc. SPIE 10056, 100560J (2017). 10.1117/12.2255867

[49] Lakowicz J. R. Keating-Nakamoto S., “Red-edge excitation of fluorescence and dynamic properties of proteins and membranes,” Biochemistry 23(13), 3013–3021 (1984). BICHAW 0006-2960 10.1021/bi00308a026 6466628 PMC6906601

[r50] Demchenko A. P., “The red-edge effects: 30 years of exploration,” Luminescence 17(1), 19–42 (2002). 10.1002/bio.671 11816059

[r51] Kabir M. L. Wang F. Clayton A. H. A., “Red-edge excitation shift spectroscopy (REES): application to hidden bound states of ligands in protein–ligand complexes,” Int. J. Mol. Sci. 22(5), 2582 (2021).1422-0067 10.3390/ijms22052582 33806656 PMC7961384

[r52] Warrender A. K. et al., “Red edge excitation shift spectroscopy is highly sensitive to tryptophan composition,” J. R. Soc. Interface 20(208), 20230337 (2023).1742-5689 10.1098/rsif.2023.0337 37935360 PMC10645072

[r53] Catici D. A. M. et al., “The red edge excitation shift phenomenon can be used to unmask protein structural ensembles: implications for NEMO–ubiquitin interactions,” FEBS J. 283(12), 2272–2284 (2016).1742-464X 10.1111/febs.13724 27028374

[r54] Lakowicz J. R., “On spectral relaxation in proteins,” Photochem. Photobiol. 72(4), 421–437 (2007). 10.1562/0031-8655(2000)0720421OSRIP2.0.CO2 PMC6906610 11045710

[55] Chattopadhyay A., “Exploring membrane organization and dynamics by the wavelength-selective fluorescence approach,” Chem. Phys. Lipids 122(1-2), 3–17 (2003). CPLIA4 0009-3084 10.1016/S0009-3084(02)00174-3 12598034

[56] DaCosta R. S. Andersson H. Wilson B. C., “Molecular fluorescence excitation-emission matrices relevant to tissue spectroscopy,” Photochem. Photobiol. 78(4), 384–392 (2007). PHCBAP 0031-8655 10.1562/0031-8655(2003)0780384MFEMRT2.0.CO2 14626667

[57] LaRochelle E. P. M. et al., “3D-printed tumor phantoms for assessment of in vivo fluorescence imaging analysis methods,” Mol. Imaging Biol. 25(1), 212–220 (2023). 10.1007/s11307-022-01783-5 36307633 PMC9970939

[58] Reindl S. et al., “Quantum yield of triplet formation for indocyanine green,” J. Photochem. Photobiol. Chem. 105(1), 65–68 (1997). 10.1016/S1010-6030(96)04584-4

[59] KARGER, “5 human albumin,” Transfus. Med. Hemother 36(6), 399–407 (2009). 21245971 PMC2997295

[60] Choi S. et al., “A rapid, simple measurement of human albumin in whole blood using a fluorescence immunoassay (I),” Clin. Chim. Acta 339(1-2), 147–156 (2004). 10.1016/j.cccn.2003.10.002 14687905

[61] Prahl S. A. van Gemert M. J. C. Welch A. J., “Determining the optical properties of turbid media by using the adding–doubling method,” Appl. Opt. 32(4), 559–568 (1993). APOPAI 0003-6935 10.1364/AO.32.000559 20802725

[62] Gijbels I. Prosdocimi I., “Loess,” WIREs Comput. Stat. 2(5), 590–599 (2010). 10.1002/wics.104

[63] Cleveland W. S. Devlin S. J., “Locally weighted regression: an approach to regression analysis by local fitting,” J. Amer. Stat. Assoc. 83(403), 596–610 (1988). JSTNAL 0003-1291 10.1080/01621459.1988.10478639

[64] Python Software Foundation, “PyPI: the Python package index,” https://pypi.org/ (accessed 20 July 2026).

[65] Cappellari M., “loess: LOESS: smoothing via robust locally-weighted regression in one or two dimensions,” Python.

[66] Cappellari M. et al., “The ATLAS3D project - XX. Mass-size and mass-σ distributions of early-type galaxies: bulge fraction drives kinematics, mass-to-light ratio, molecular gas fraction and stellar initial mass function,” Mon. Not. R. Astron. Soc. 432, 1862–1893 (2013). MNRAA4 0035-8711 10.1093/mnras/stt644

[67] Panigrahi S. K. Mishra A. K., “Inner filter effect in fluorescence spectroscopy: as a problem and as a solution,” J. Photochem. Photobiol. C Photochem. Rev. 41, 100318 (2019). 10.1016/j.jphotochemrev.2019.100318

[r68] Lakowicz J. R., “Quenching of fluorescence,” in Principles of Fluorescence Spectroscopy, Lakowicz J. R., Ed., pp. 257–301, Springer US, Boston, MA (1983).

[69] Nguyen M. H. et al., “Toward fluorescence digital twins: multi-parameter experimental validation of fluorescence Monte Carlo simulations using solid phantoms,” J. Biomed. Opt. 30(S3), S34104 (2025). JBOPFO 1083-3668 10.1117/1.JBO.30.S3.S34104 40443945 PMC12119851

[70] Bai L. et al., “Super-stable cyanine@albumin fluorophore for enhanced NIR-II bioimaging,” Theranostics 12(10), 4536–4547 (2022). 10.7150/thno.71443 35832086 PMC9254253

[71] Rotermund F. et al., “Fluorescence spectroscopic analysis of indocyanine green J aggregates in water,” J. Photochem. Photobiol. Chem. 110(1), 75–78 (1997). 10.1016/S1010-6030(97)00167-6

[r72] Luciani X. Redon R. Mounier S., “How to correct inner filter effects altering 3D fluorescence spectra by using a mirrored cell,” Chemom. Intell. Lab. Syst. 126, 91–99 (2013). 10.1016/j.chemolab.2013.04.014

[73] Murphy K. R. et al., “Fluorescence spectroscopy and multi-way techniques. PARAFAC,” Anal. Methods 5(23), 6557 (2013). AMNEGX 1759-9679 10.1039/c3ay41160e

[74] Ellmerer M. et al., “Measurement of interstitial albumin in human skeletal muscle and adipose tissue by open-flow microperfusion,” Amer. J. Physiol.-Endocrinol. Metab. 278(2), E352–E356 (2000). 10.1152/ajpendo.2000.278.2.E352 10662720

[75] Harris A. T., “Spectral mapping tools from the earth sciences applied to spectral microscopy data,” Cytom. Part A 69A, 872–879 (2006). 10.1002/cyto.a.20309 16969808

[76] Rossetti B. J. et al., “Semi-blind sparse affine spectral unmixing of autofluorescence-contaminated micrographs,” Bioinformatics 36(3), 910–917 (2020). BOINFP 1367-4803 10.1093/bioinformatics/btz674 31504202 PMC7523684

[77] Davis S. C. et al., “Spectral distortion in diffuse molecular luminescence tomography in turbid media,” J. Appl. Phys. 105(10), 102024 (2009). JAPIAU 0021-8979 10.1063/1.3116130 20157444 PMC2821414

[78] Wang T. Zeng L.-H. Li D.-L., “A review on the methods for correcting the fluorescence inner-filter effect of fluorescence spectrum,” Appl. Spectrosc. Rev. 52(10), 883–908 (2017). APSRBB 0570-4928 10.1080/05704928.2017.1345758

